# Cholera in Calcutta

**Published:** 1861-07

**Authors:** 


CHOLERA IN CALCUTTA.—From accounts recently received in Europe and this country, it appears that this disease is now raging in a frightful degree in Calcutta and its suburbs.

